# Next-Generation Sequencing Enhances the Diagnosis Efficiency in Thyroid Nodules

**DOI:** 10.3389/fonc.2021.677892

**Published:** 2021-07-12

**Authors:** Li-Cheng Tan, Wan-Lin Liu, Xiao-Li Zhu, Peng-Cheng Yu, Xiao Shi, Pei-Zhen Han, Ling Zhang, Liang-Yu Lin, Arseny Semenov, Yu Wang, Qing-Hai Ji, Dong-Mei Ji, Yu-Long Wang, Ning Qu

**Affiliations:** ^1^ Department of Head and Neck Surgery, Fudan University Shanghai Cancer Center, Shanghai, China; ^2^ Department of Oncology, Shanghai Medical College, Fudan University, Shanghai, China; ^3^ Department of Pathology, Fudan University Shanghai Cancer Center, Shanghai, China; ^4^ Department of Technology, Zhejiang Topgen Clinical Laboratory Co, Ltd., Huzhou, China; ^5^ Endocrine Surgery Department, N.I. Pirogov Clinic of High Medical Technologies, Saint-Petersburg State University, Saint-Petersburg, Russia; ^6^ Department of Medical Oncology, Fudan University Shanghai Cancer Center, Shanghai, China

**Keywords:** thyroid nodules, next-generation sequencing, fine-needle aspiration, diagnosis, BRAF mutation

## Abstract

**Background:**

Though fine-needle aspiration (FNA) improved the diagnostic methods of thyroid nodules, there are still parts of nodules that cannot be determined according to cytology. In the Bethesda system for reporting thyroid cytopathology, there are two uncertain cytology results. Thanks to the development of next-generation sequencing technology, it is possible to gain the genetic background of pathological tissue efficiently. Therefore, a combination of the cytology and genetic background may enhance the accuracy of diagnosis in thyroid nodules.

**Methods:**

DNA from 73 FNA samples of thyroid nodules belonging to different cytology types was extracted and exome sequencing was performed by the ThyroLead panel. Test for BRAF mutation was also performed by ARMS-qPCR. Information including age, sex, preoperative cytology, *BRAF* mutation status tested by ARMS-qPCR, and surgical pathology was collected in electronic medical record system.

**Results:**

A total of 71 single nucleotide variants, three fusion gene, and two microsatellite instability-high status were detected in 73 FNA samples. BRAF V600E mutation is the most common mutation in these malignant thyroid nodules. After combining the cytology and genetic background detected by next-generation sequencing, the diagnosis sensitivity was increased from 0.582 (95% CI: 0.441–0.711) to 0.855 (95% CI: 0.728–0.930) (*P* < 0.001) in our group, while the specificity, 1,000 (95% CI: 0.732–1.000) compared to 0.857 (95% CI: 0.562–0.975) (*P* = 0.25), did not get affected.

**Conclusions:**

Next-generation sequencing in thyroid nodules can enhance the preoperative diagnosis sensitivity by fine-needle aspiration alone. It can also provide genetic background for direction of medication. It is possible for clinicians to combine cytology with genetic alterations for a more precise diagnosis strategy of thyroid nodules.

## Introduction

Thyroid cancer came to the 11th place of most common cancer worldwide and 5th in adult women (1), ranking first among endocrine malignancies. Papillary thyroid carcinoma (PTC) and follicular thyroid carcinoma (FTC) known as differentiated thyroid carcinoma (DTC), contribute to more than 95% thyroid malignancies (2). This dramatic increase in the incidence of these cancers is partly due to the improvement of screening methods, such as high-resolution ultrasonic scanning and fine-needle aspiration (FNA) cytopathology, for detecting subclinical thyroid cancers (3). Though thyroid cancer has favorable overall survival, early and accurate diagnosis is crucial for treatment and disease management (4).

Almost all thyroid neoplasms behave as thyroid nodules when first examined, and FNA can provide a robust and rapid evidence to define the characteristics of doubtful nodules after ultrasonic scanning (5). The Bethesda System for Reporting Thyroid Cytopathology (TBSRTC) contains two uncertain cytology between the benign and malignant types. One is Bethesda category III, which contains Atypia of Undetermined Significance (AUS) and Follicular Lesion of Undetermined Significance (FLUS), and another is Bethesda category IV containing Follicular Neoplasm (FN) and Suspicious for a Follicular Neoplasm (SFN) (6). The frequency of incidence of the two uncertain types cytology can reach about 10% respectively (5), leading to a dilemma for both clinicians and patients.

To cope with this predicament, some studies proposed strategies that combine cytopathology with the molecular pattern of aspiration samples to enhance the accuracy of diagnosis (7–9). However, to the best of our knowledge, no research has focused on the difference in genetic change between the nodule of Bethesda III or IV and malignant nodules. Therefore, in this study, we selected 73 samples from patients with thyroid nodules of Bethesda II to VI who underwent FNA in our center to perform target sequencing of thyroid cancer-related genes.

## Materials and Methods

### Sample Collection and Disposal

We first retrospectively searched FNA samples recorded from 2016 to 2020 and then collected 73 snap-frozen tissue of FNA samples of thyroid nodules at the Department of Pathology, Fudan University Shanghai Cancer Center. DNA was isolated using QIAamp DNA kit (Qiagen, Dusseldorf, Germany).

### ARMS-qPCR for *BRAF* Mutation

*BRAF* mutation detection assays were routinely performed in our center using *BRAF* V600E Diagnostic Kit (AmoyDx, China) 145 on an ABI 7500 Real-time PCR system (Life Technologist, 146 USA) according to the instructions of the manufacturer. Genomic DNA, 5–10 ng, was added in a 50 μl system in each assay. The 147 amplification conditions included: 1 cycle at 95°C for 5 min, 15 cycles at 95°C for 25 s, 64°C for 20 s, and 72°C for 20 s, 31 cycles at 93°C for 25 s, 60°C for 35 s, and 72°C for 20 s, and the fluorescent signals were recorded at 60°C. 149 mutation status classification was identified following the manuscripts of kits.

### Next-Generation Sequencing

For targeted next-generation sequencing analysis, the ThyroLead panel (Topgen, China) covers the coding exons of 16 genes (*BRAF*, *HRAS*, *KRAS*, *NRAS*, *RET*, *PIK3CA*, *CTNNB1*, *TP53*, *PTEN*, *IDH1*, *DICER1*, *MEN1*, *MTOR*, *TSHR*, *CDC73*, *CDKN1B*), a 1,000-bp region in the Promoter region of *TERT*, and both coding exons and 12 introns of four frequently rearranged genes(*BRAF*, *NTRK1*, *RET* and *PPARG*). At least 30 ng genomic DNA was added and sheared using the Covaris E220 instrument (Covaris, Woburn, MA). Sequence libraries were prepared using KAPA HyperPlus Library Preparation Kit by first producing blunt-ended, 5’-phosphorylated fragments. To the 3’ ends of the dsDNA library fragments, dAMP was added (A-tailing). Next, dsDNA adapters with 3’-dTMP were ligated to the A-tailed library fragments. Library fragments with appropriate adapter sequences were amplified *via* ligation-mediated pre-capture PCR. Library capture was conducted using Igenetech custom probes system and were biotinylated to allow for sequence enrichment by capture using streptavidin-conjugated beads (Thermo Fisher). Pooled libraries containing captured DNA fragments were subsequently sequenced using the NextSeq 500/550 High Output Kit v2.5 (300 cycles) on the Illumina NextSeq 500 system as 2 × 150-bp paired-end reads.

### Data Analysis

Illumina software bcl2fastq was used to generate FastQ files; the number of mismatches was set to 1. The generated FastQ files were processed by software Fastp to remove adaptor sequences as well as poor-quality reads (with more than 40% of bases have phred-scale quality of less than 15) (10). Remaining reads were mapped to hg19 reference genome using BWA-MEM algorithm (11), followed by sorting and duplicate-marking using SAMtools v1.9 and Picard v1.76 respectively. Next, commercial software Sentieon TNseq (version 20180808) was adopted to infer short somatic variants with default parameters (12). Software GeneFuse was used to detect fusions (13). All reported variants were manually checked on IGV to ensure their reliability (14). ANNOVAR (2019Oct24) was used to annotate reported SNVs and small InDels (15). As germline sequencing data were not available, we adopted a strict filtering strategy to remove potential germline variants (16). Variants presented in one or more population databases (ESP, 1000Genome, ExAC, gnomAD) with MAF >= 1% were excluded (17–20). Variants listed as “benign” in the ClinVar database were discarded (21).

Sequence variant confirmation was performed by conventional techniques including Sanger sequencing and real-time PCR. The calculation of analytical accuracy, limits of detection, and assay reproducibility was performed using MedCalc Statistical Software version 9.6. The waterfall plot of SNVs was drawn by R package GenVisR (22).

### Statistical Analysis

Fisher’s exact test for categorical variables in paired fourfold table was used. A *P*-value <0.05 was considered statistically significant.

## Results

### Basic Information of the Patients Included in This Study

Clinical and demographic data of the patients whose samples were collected for this study were retrieved from the electronic medical record system of our institution. Data on sex, age, Bethesda cytology, and surgical pathology of the study population are provided in **Table 1**. Among the 73 samples, 14, 27, and 32 were classified as Bethesda II, III/IV, and V/VI respectively. A total of 69 out of 73 patients received surgery in our center and 55 were diagnosed as malignant thyroid tumor, including 53 PTC, 1 FTC, and 1 medullary thyroid tumor (MTC).

**Table 1:** Basic information of patients involed in this study.

Information of the patients (n = 73)		
Sex	Male	14 (19.18%)
	Female	59 (80.82%)
Age of FNA		42.96±11.96
Cytology	Bethesda II	14 (19.18%)
	Bethesda III/IV	27 (36.99%)
	Bethesda V/VI	32 (43.83%)
Surgical pathology	PTC	53 (72.60%)
	FTC	1 (1.37%)
	MTC	1 (1.37%)
	Benign	14 (19.18%)
	No surgery	4 (5.48%)

Sex, age, preoperative cytology, and surgical pathology are listed.

### Next-Generation Sequencing of FNA Samples

**Figure 1:**
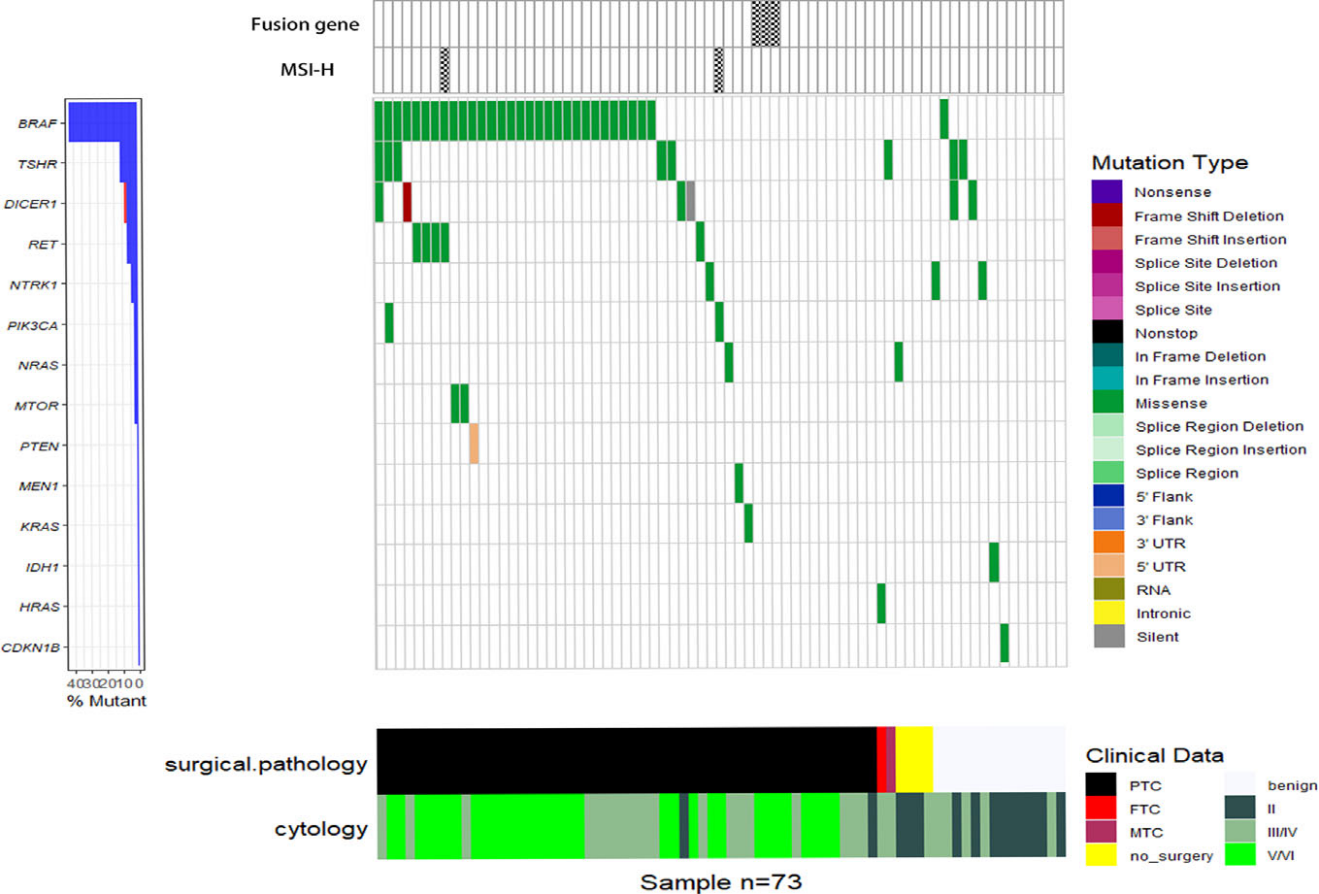
Waterfall plot of SNVs, fusion genes, and MSI-H status detected by NGS. Various types of SNVs, fusion genes and MSI-H status of 73 FNA samples (each vertical column represents a sample) tested by the ThyroLead panel are displayed and the corresponding cytology/pathology of each sample was listed in the bottom. Mutations of different types are marked with respective colors and the spoted grids on the top mean positive fusion genes or MSI-H status.

All the FNA samples were tested with the ThyroLead for target sequencing (see details in *Methods and Materials*), and all quality control (QC) information was listed in **Supplementary Table S2**. Mutation mapping of single nucleotide variants, fusion genes, and microsatellite instability status (MSI) were shown in **Figure 1**. A total of 71 single nucleotide mutations were detected unsing ThyroLead (**Table 2**). *BRAF* V600E was the most common mutation in thyroid cancer (23), and it all occurred in Bethesda III or higher grade nodules which were categorized by TBSRTC. *RET* fusion genes, another hallmarker in BRAF-like thyroid cancer (24), were detected in three Bethesda V/VI nodules, and all of these nodules were diagnosed as thyroid cancer after surgery. In addition, four types of *RET* single nucleotide mutations with uncertain significance were found in Bethesda III/IV and Bethesda V/VI nodules. NRAS, KRAS, and HRAS mutations were found in two, one, and one sample(s) respectively. Three of these four patients underwent surgery, and their samples were determined to be malignant.

**Table 2:** Genetic alterations of 73 samples.

Genetic alterations											
Alteration types			Group A (Bethesda II, n=14)		Group B (Bethesda, III/IV n=27)		Group C (Bethesda, V/VI n=32)		Surgical pathology		
			number of alterations	frequency	number of alterations	frequency	number of alterations	frequency	benign	malignant	no surgery
**Single nucleotide mutation**	***BRAF***	BRAF Val600Glu	0	0.00%	12	44.44%	19	59.38%	1	30	0
	***TSHR***	TSHR Arg450His	1	7.14%	0	0.00%	0	0.00%	1	0	0
		TSHR Thr632Ile	0	0.00%	1	3.70%	0	0.00%	1	0	0
		TSHR Ile591Val	0	0.00%	1	3.70%	0	0.00%	0	1	0
		TSHR Ala275Thr	0	0.00%	1	3.70%	0	0.00%	0	1	0
		TSHR Lys700Glu	0	0.00%	0	0.00%	1	3.13%	0	1	0
		TSHR Val689Gly	0	0.00%	0	0.00%	1	3.13%	0	1	0
		TSHR Phe525Ser	0	0.00%	0	0.00%	1	3.13%	0	1	0
		TSHR Ser305Arg	0	0.00%	0	0.00%	1	3.13%	0	1	0
	***DICER1***	DICER1 Asp1810Val	1	7.14%	0	0.00%	0	0.00%	0	1	0
		DICER1 Asp1709Asn	1	7.14%	0	0.00%	0	0.00%	1	0	0
		DICER1 Asp619Gly	1	7.14%	0	0.00%	0	0.00%	1	0	0
		DICER1 Thr453Ser	0	0.00%	1	3.70%	0	0.00%	0	1	0
		DICER1 Asp1709Glu	0	0.00%	1	3.70%	0	0.00%	0	1	0
		*DICER1* c.3243del	0	0.00%	1	3.70%	0	0.00%	0	1	0
		DICER1 Lys191=	0	0.00%	0	0.00%	1	3.13%	0	1	0
	***RET***	RET Val292Met	0	0.00%	1	3.70%	0	0.00%	0	1	0
		RET Arg67His	0	0.00%	1	3.70%	3	9.38%	0	4	0
		RET Arg982Cys	0	0.00%	1	3.70%	3	9.38%	0	4	0
		RET Arg982His	0	0.00%	0	0.00%	1	3.13%	0	1	0
	***NTRK1***	NTRK1 Ser396Leu	0	0.00%	1	3.70%	0	0.00%	1	0	0
		NTRK1 Arg273Gln	0	0.00%	1	3.70%	0	0.00%	1	0	0
		NTRK1 Asn323Ser	0	0.00%	0	0.00%	1	3.13%	0	1	0
	***PIK3CA***	PIK3CA His1047Tyr	0	0.00%	0	0.00%	1	3.13%	0	1	0
		PIK3CA Pro316Thr	0	0.00%	0	0.00%	1	3.13%	0	1	0
	***NRAS***	NRAS Gln61Arg	1	7.14%	1	3.70%	0	0.00%	0	1	1
	***MTOR***	MTOR Ala1792Val	0	0.00%	1	3.70%	0	0.00%	0	1	0
		MTOR Ile1973Phe	0	0.00%	0	0.00%	1	3.13%	0	1	0
	***MEN1***	MEN1 Arg176Gln	0	0.00%	1	3.70%	0	0.00%	0	1	0
	***PTEN***	*PTEN* c.-156C>G	0	0.00%	0	0.00%	1	3.13%	0	1	0
	***KRAS***	KRAS Gln61Lys	0	0.00%	1	3.70%	0	0.00%	0	1	0
	***IDH1***	IDH1 Tyr208Cys	1	7.14%	0	0.00%	0	0.00%	1	0	0
	***HRAS***	HRAS Gln61Lys	0	0.00%	1	3.70%	0	0.00%	0	1	0
	***CDKN1B***	CDKN1B Glu75Asp	1	7.14%	0	0.00%	0	0.00%	1	0	0
		Total	7		28		36		9	61	1
**Fusion gene**	***RET***	NCOA4 exon7-RET exon12	0	0.00%	0	0.00%	1	3.13%	0	1	0
		CCDC6 exon1-RET exon11	0	0.00%	0	0.00%	1	3.13%	0	1	0
		RET exon11-CCDC6 exon2	0	0.00%	0	0.00%	1	3.13%	0	1	0
**MSI**		MSI-H	0	0.00%	0	0.00%	2	6.25%	0	2	0

Specific points of SNVs, fusion genes, and MSI status are listed in accordance with the sequence of cytology and pathology types.

Eight different types of *TSHR* mutations were identified. The *TSHR* Thr632Ile, which was previously detected in thyroid cancer with uncertain significance (25–27), occurred in one Bethesda III/IV nodule. However, it was found to be associated with a benign lesion. The other seven undefined *TSHR* mutations were sporadically present in various types of cytology.

Mutation of the tumor suppressor gene, *DICER1*, is related to DICER1 syndrome, which often manifests in an autosomal dominant manner (28, 29). Different from most tumor suppressor genes, loss of one *DICER1* allele is sufficient to induce tumorgenesis (30), while homozygous deletion is rare in *DICER1*-related diseases (31). Furthermore, direct correlation between *DICER1* mutations and thyroid cancer has been reported previously (32, 33). Three pathogenic substitutions (two in D1709, one in D1810) and one frameshift deletion of *DICER1* were found among the three samples, and two of them were verified as PTC by surgical pathology. However, we were unable to ascertain whether the alterations were germline mutations due to a lack of blood samples.

MSI results from defects in the DNA mismatch repair system (MMR) (34), which is related to a wide range of types, including thyroid cancer (35–37). Through NGS, we detected two samples with MSI-high (MSI-H) status, and they were determined to be papillary thyroid microcarcinoma through surgical pathology.

In addition, pathogenic mutations, including *PIK3CA* H1047 (38, 39), and mutations with uncertain significance including *NTRK1*, *MTOR*, *PTEN*, *IDH1*, *MEN1*, *PIK3CA*, and *CDKN1B* were detected. The co-existence of *BRAF* V600E and *DICER1* D1709E along with *DICER1* frameshift deletion, *BRAF* V600E with *PIK3CA* H1047Y, and *BRAF* V600E with MSI-H was found in three samples, respectively.

### ARMS-qPCR Was More Sensitive Than NGS in *BRAF* V600E Detection

We have reported that ARMS-qPCR has a higher sensitivity than Sanger sequencing in detecting the *BRAF* V600E mutation (40), but the efficiency of NGS in *BRAF* V600E detection was unknown. Therefore, we compared the *BRAF* V600E mutation frequencies, which were determined by ThyroLead and ARMS-qPCR in our samples. We found that BRAF V600E mutation was detected in 39 of 73 (53.42%) samples and 31 of 73 (42.47%) samples by ARMS-qPCR and NGS, respectively (**Supplementary Table S1**), and all the positive samples tested by NGS were also positive by ARMS-qPCR. Therefore, the sentivity of ARMS-qPCR in detecting *BRAF* V600E mutation was higher than that of NGS.

### Combination of Cytology With NGS Improved the Rate of Detection of Malignant Nodules

To determine the diagnostic efficiency of NGS for malignant nodules, we compared the diagnostic sensitivity and specificity of cytology, along with a combination of cytology and NGS. Among the 73 patients involved in this study, 69 patients received thyroidectomy and got final surgical pathology. After combining cytology with NGS data, diagnostic sensitivity increased from 0.582 (0.441–0.711) to 0.855 (0.725–0.930) (*P* < 0.001), while specificity did not show statistically significant change (*P* = 0.25, **Tables 3**, **4**). Thus, we concluded that the combination of cytology and NGS can markedly improve the detection rate of malignant thyroid nodules compared to that by cytology alone.

**Table 3:** Diagnostic efficiency of cytology and cytology combined with NGS.

Diagnosis specificity and sensitivity			
**Cytology**			
	Bethesda V/VI	Bethesda II to IV	
Malignant	32	23	sensitivity with 95% CI: 0.582 (0.441–0.711)
Benign	0	14	specificity with 95% CI: 1.000 (0.732–1.000)
**NGS with cytology**			
	Bethesda V/VI or (with) pathogenic mutation	Bethesda II to IV without pathogenic mutation	
Malignant	47	8	sensitivity with 95% CI: 0.855 (0.728–0.930)
Benign	2	12	specificity with 95% CI: 0.857 (0.562–0.975)

Sensitivity and specificity of diagnosis based on cytology (Bethesda V/VI are considered malignant) and combination of cytology and NGS (Bethesda V/VI and/or pathogenic mutation are considered malignant).

**Table 4:** Improvement by combining cytology with NGS in diagnosis.

Comparison of diagnosis efficiency				
Malignant (n = 55)		Cytology		
		+	-	
NGS with cytology	+	32	15	*P* < 0.001
	–	0	8	
Benign (n = 14)		Cytology		
		+	–	
NGS with cytology	+	0	2	*P* = 0.25
	–	0	12	

Comparison of the diagnosis efficiency of cytology along or with NGS.

### Diagnostic Efficiency of Combined Data of ARMS-qPCR and Genetic Alterations

Although *BRAF* V600E has a favorable diagnostic value in thyroid cancer (23), it only provides evidence for PTC or PTC-derived poorly differentiated thyroid carcinoma (41). To explore whether NGS can increase the accuracy of diagnosis compared with methods based on the detection of *BRAF* mutations, we analyzed the diagnostic specificity and sensitivity of thyroid cancer by FNA cytology combined with ThyroLead or with ARMS-qPCR for *BRAF* mutation merely. As to combination of cytology with ARMS-qPCR, the diagnotic sensitivity and specificity were 0.873 (95% CI: 0.749–0.943) and 0.857 (95% CI: 0.562–0.975), respectively (**Table 5**) and showed no statistical significance compared to that obtained by combination of cytology and NGS (**Table 6**). Four thyroid carcinomas with wildtype *BRAF* were classified as malignant nodules by NGS, and five *BRAF* V600E mutations were detected by ARMS-qPCR and missed by NGS.

**Table 5:** Diagnosis efficiency of combined diagnostic approaches.

Diagnosis specificity and sensitivity			
**ARMS-qPCR with cytology**			
	Bethesda V/VI or (with) BRAF mutation	Bethesda II to IV with WT BRAF	
Malignant	48	7	sensitiviry with 95% CI: 0.873 (0.749-0.943)
Benign	2	12	specificity with 95% CI: 0.857 (0.562-0.975)
**ARMS-qPCR and NGS with cytology**			
	Bethesda V/VI or (with) pathogenic mutation	Bethesda II to IV without pathogenic mutation	
Malignant	52	3	sensitiviry with 95% CI: 0.945 (0.839-0.986)
Benign	3	11	specificity with 95% CI: 0.785 (0.488-0.943

Sensitivity and specificity of diagnosis based on combination of cytology and ARMS-qPCR for BRAF V600E mutation detection (Bethesda V/VI with/or BRAF mutation are considered malignant) and combination of cytology, ARMS-qPCR and NGS (Bethesda V/VI and/or pathogenic mutation are considered malignant).

**Table 6:** Comparison of diagnosis efficiency of three combined diagnostic approaches.

Comparison of diagnosis efficiency				
Malignant (n = 55)		ARMS-qPCR with cytology		
		+	-	
NGS with cytology	+	43	4	*P* = 0.5
	–	5	3	
Benign (n = 14)		ARMS-qPCR with cytology		
		+	–	
NGS cytology	+	1	1	*P* = 0.75
	–	1	11	
Malignant (n = 55)		ARMS-qPCR and NGS with cytology		
		+	–	
NGS with cytology	+	47	0	*P* = 0.0313
	–	5	3	
Benign (n = 14)		ARMS-qPCR and NGS with cytology		
		+	–	
NGS with cytology	+	2	0	*P* = 0.5
	–	1	11	
Malignant (n = 55)		ARMS-qPCR and NGS with cytology		
		+	–	
ARMS-qPCR with cytology	+	48	0	*P* = 0.0625
	–	4	3	
Benign (n = 14)		ARMS-qPCR and NGS with cytology		
		+	–	
ARMS-qPCR with cytology	+	2	0	*P* = 0.5
	–	1	11	

We then combined the ARMS-qPCR and specific pathogenic mutations obtained by ThyroLead and recalculated the diagnostic sensitivity and specificity. We found that 52 of the 55 cancerous nodules showed malignant cytology characteristics or/with pathogenic mutation, with a sensitivity of 0.9454 (95% CI: 0.839–0.986) and the specificity of 0.785 (95% CI: 0.488–0.943) (**Table 5**).

The sensitivity of the combination group was higher than that of the NGS group (*P* = 0.0313) but showed no statistical significance compared with the ARMS-qPCR group (*P* = 0.0625). The specificity between the three groups did not differ significantly (*P* = 0.5). Comparison of the diagnotic efficiencies is shown in **Table 6**.

## Discussion

Standard and advanced preoperative thyroid biopsy methods, which enable patients to receive optimal treatment and avoid unnecessary surgery, have been employed for early and precise diagnosis of thyroid cancer (42). Nonetheless, a considerable proportion of nodules remain ambiguous after FNA, necessitating the need for more accurate diagnostic approaches. *BRAF* V600E is the earliest and most widely used molecular marker for PTC diagnosis (43). However, there are no such established molecular markers for the remaining pathological types of thyroid cancer, especially FTC (44, 45), often complicating the preoperative diagnosis. Moreover, the prognosis of these non-PTC thyroid cancers is commonly worse because of severe local invasion or distant metastasis (46). This means that it is challenging to diagnose highly malignant cancers preoperatively. NGS technology has revealed genetic alterations underlying thyroid cancer with high efficiency (47). The findings of this study also revealed increase in sensitivity of diagnosis by using NGS to detect cancer-related mutation in thyroid FNA samples including non-PTC cancers.

The Cancer Genome Atlas (TCGA) Research Network described the genomic landscape of PTC in 2014 (24) and revealed the most comprehensive genetic alteration behind PTC. In this large sample atlas, 3.5% of the tumor samples lacked obvious cancer drivers (point mutations, fusion genes, and somatic copy number alterations). In our group, nine of 53 (16.98%) PTC patients showed no cancer drivers (point mutations, fusion genes, and MSI-H), indicating that a more comprehensive target panel could be taken into consideration to enhance the sensitivity of diagnosis.

According to previous reports, *BRAF* V600E is rarely found in benign thyroid lesion (48). Incidentally, two nodules with *BRAF* mutations and AUS cytology were surgically proven to be benign in this study. The pathological features represented Hashimoto thyroiditis with dysplasia in individual follicular epithelium for one and adenomatous nodules for another. In addition, the pathogenic TSHR Thr632Ile (25, 26) mutation was found in one sample of AUS cytology, and the final pathology indicated a nodular goiter with fibrosis and calcification. It is unclear whether this group of nodules with pathogenic mutations but benign pathological features is precancerous or just benign lesions. Thus, further studies on the transformation of normal epithelium to a malignant one driven by pathogenic mutations are warranted.

DICER1 functions as an enzyme, which participates in the production of miRNAs (49). Most frequently, germline loss-of-function mutations in *DICER1* combined with a somatic mutation in hotspot (D1705, D1709, G1809, D1810, and E1813) of the RNase IIIb region contribute to the DICER1 syndrome, manifesting as susceptibility to various tumors (50). Plenary studies have shown a correlation between *DICER1* syndrome and thyroid cancer (32, 33), and studies revealed that the loss expression of *DICER1* was correlated with the malignant status of thyroid cancer cells in a miRNA-dependent manner both *in vitro* and *in vivo*, and predicted a worse outcome in patients with thyroid cancers (51, 52); however, whether a mere somatic mutation can lead to thyroid cancer is uncertain.

We hypothesize that the development of NGS technology in diagnosis will lead to the discovery of more nodules, which can advance our understanding of thyroid tumorigenesis.

Although ARMS-qPCR is more sensitive in detecting *BRAF* V600E than Sanger (40) and NGS, the result is restricted to a single mutation. Evidence has shown that the co-existence of mutations, including *BRAF*, *TERET*, and *P53* often predicts poor prognosis in thyroid cancer (53). It is crucial to identify this group of patients to provide early and personalized treatment, which may improve their outcomes. NGS has the capacity to detect multiple mutations simultaneously, aiding clinicians to perform early interventions in patients with worse prognosis due to the co-existence of mutations. The combination of ARMS-qPCR and NGS showed an increase in diagnostic sensitivity, although it did not reach statistical significance when compared to ARMS-qPCR alone, which may be a result of the limited sample size.

The NGS data of FNA provides not only clues for diagnosis, but also the direction for medication. The BRAF inhibitor dabrafenib and MEK inhibitor trametinib have achieved great success in *BRAF* V600E-mutated anaplastic thyroid carcinoma (ATC) (54). Targeted therapy for other mutations also showed a breakthrough in advanced thyroid cancer (55, 56). Recent studies have shown that several solid tumors with MSI-H status showed good response to PD-1 inhibitors (57–59) and the efficacy of the immune checkpoint inhibitor (ICI) in ATC has been proven (60). Thus, MSI status could be a potential predictive factor for immunotherapy in advanced thyroid cancer.

Our study had some limitations. First, due to the small number of samples in our study, we were unable to associate some mutations with benign or malignant thyroid nodules. Moreover, some potentially instructive mutations were not detected in NGS sequencing. Second, we were unable to evaluate the germline mutation of DICER1. Therefore, further studies with a more complete panel are warranted.

## Conclusions

In this study, we performed exome sequencing of FNA samples of thyroid nodules belonging to different cytology types and mapped mutations in the corresponding patients. By analyzing the sequencing data between preoperative cytology types and final pathology after surgery, we concluded that the combination of cytology with genetic alterations contributes to a more precise diagnosis strategy for thyroid nodules.

## Data Availability Statement

The data are now openly accessible in China National Center for Bioinformation at https://bigd.big.ac.cn/gsa-human/browse/HRA000846.

## Ethics Statement

The studies involving human participants were reviewed and approved by the Ethics Committee of Fudan University Shanghai Cancer Center. The patients/participants provided their written informed consent to participate in this study.

## Author Contributions

Conceptualization, NQ and Y-LW. Methodology, D-MJ and L-YL. Writing-original draft preparation, L-CT and W-LL. Investigation, W-LL, L-CT, and LZ. Visualization, L-CT and P-ZH. Writing—review and editing, Y-LW, NQ, D-MJ, YW, and Q-HJ, and AS. Data curation, L-CT, X-LZ, and L-YL. Resources, X-LZ, P-CY, and XS. Supervision, D-MJ. Project administration, NQ and Y-LW. Funding acquisition, NQ and Y-LW. All authors contributed to the article and approved the submitted version.

## Funding

This work was supported by the National Natural Science Foundation of China (82002831 to W-LL, 81772851 and 81972501 to Y-LW, 81702649 to NQ).

## Conflict of Interest

Author L-YL was employed by Zhejiang Topgen Clinical Laboratory Co, Ltd.

The remaining authors declare that the research was conducted in the absence of any commercial or financial relationships that could be construed as a potential conflict of interest.
